# Humour interventions for patients in palliative care—a randomized controlled trial

**DOI:** 10.1007/s00520-023-07606-9

**Published:** 2023-02-13

**Authors:** Lisa Linge-Dahl, Rainer Kreuz, Mieke Stoffelen, Sonja Heintz, Willibald Ruch, Eckart von Hirschhausen, Lukas Radbruch

**Affiliations:** 1 Department of Palliative Medicine, University of Bonn, Bonn, Germany; 2 Foundation “Humor Hilft Heilen” (Humour Helps to Cure), Bonn, Germany; 3 Department of Psychology, University of Zurich, Zurich, Switzerland; 4 School of Psychology, University of Plymouth, Plymouth, UK; 5 Centre for Palliative Care, Helios Hospital Bonn/Rhein-Sieg, Bonn, Germany

**Keywords:** Humour, Intervention, Patient, Palliative care

## Abstract

**Purpose:**

The effect of humour on end-of-life patients could be beneficial and is worth investigating. However, data on humour interventions for patients in palliative care are scarce. This study evaluated the effects of a humour intervention in a palliative care setting.

**Methods:**

A two-step intervention was developed based on the humour habits programme by McGhee. Patients were assisted to remember funny episodes from their past and recognize humorous aspects of the present and encouraged to produce humour. The intervention and control group completed questionnaires on life satisfaction, cheerfulness, symptom burden, and perceived stress and if possible gave saliva samples to investigate oxytocin levels. The study was a randomized controlled monocentre study on patients treated in a palliative care ward. Participants had to be conscious and alert enough to complete data collection. Overall, 55 patients were included and randomized to the intervention or control group.

**Results:**

Parameters in the control group did not change significantly. In the intervention group, seriousness, bad mood, and stress were reduced. Cheerfulness increased significantly after the intervention. However, the methodologically complex intervention setting was too exhausting for the majority of patients.

**Conclusion:**

Patients who were able to participate benefited from the effects of the intervention on multiple levels. For future research simple interventions, biomarkers for well-being and assessments by staff or proxies are needed to include patients with reduced cognitive and physical performance status at the end of their lives.

**Trial registration:**

DRKS00028978 German Registry of Clinical Studies.

**Supplementary Information:**

The online version contains supplementary material available at 10.1007/s00520-023-07606-9.

## Background

Humour has been investigated in various contexts in the past, but a range of diverging definitions has been used in these studies. Ruch [1] defined the perception that something is funny as prerequisite for the occurrence of humour. Martin and Ford [2] defined humour as a broad, multifaceted term that represents anything that people say or do and that others perceive as funny and tends to make them laugh but also included thoughts and the emotional response such as enjoyment and mirth to humorous stimuli. They stated that humour essentially is a way for people to interact in a playful manner. In the expression of humour, eight comic styles have been defined [3], including lighter (fun, humour, nonsense, and wit) and darker styles (irony, satire, sarcasm, and cynicism). The darker styles were associated with a potentially negative-critical effect.

Humour interventions in patients with palliative diagnosis have rarely been implemented or systematically evaluated. Recent systematic reviews have summarized the available evidence from Pinna et al. [4] and Linge-Dahl et al. [5] showing that humour serves different purposes, such as forming relationships (e.g. between patient and health care professional) or dealing with circumstances, and have mostly been researched in health care professionals. The few studies evaluating the effect of humour from the patients’ perspective reported only unstructured qualitative data. Pinna et al. [4] also distinguished between humour before and after the diagnosis of terminal illness and emphasized that there were also situations in which humour should not be used, such as coma or when people are on the verge of death. Patients suffering from certain pneumonic illnesses such as COPD might risk hyperinflation during intensive laughter [6], so these patients should not be included in humour studies. Kontos et al. [7] studied the effects of humour interventions in dementia care homes in Australia and demonstrated reduced agitation and aggression in residents. Adamle and Ludwick [8] illustrated that humour during the interaction with the patient was also frequently observed in hospices and was mainly initiated by the patients themselves. Based on the current state of literature [4, 5, 7], no commonly used styles of humour in palliative care can be defined. We developed and pilot tested an adapted version of the five-step humour training for psychiatric patients based on McGhee [9], as this programme is supported by research and clinical applications. In addition, it comes with a well-tested manual that has been applied in various areas. Based on previous studies, we chose an outcome measure premised on the state-trait model of cheerfulness [10]. The experiences and promising results of humour studies in paediatrics [11, 12] led to the inclusion of a biomarker parameter in this study.

### Objectives

We hoped to improve the foundation of knowledge of suitable evaluation instruments for interventions in a palliative care setting. We investigated the effect of a humour intervention on life satisfaction, cheerfulness, seriousness, bad mood, symptom burden, level of stress, and oxytocin in saliva. We hypothesized that the intervention would reduce levels of stress and symptom burden and improve mood and cheerfulness in comparison to a control group without the intervention.

## Methods

### Sample/study design

We implemented a parallel study design with two groups with equal randomization. A pilot test used a more elaborate study setting with extensive questionnaires and quantitative sensory pain threshold testing [13]. As the recruitment rate was extremely poor and due to ethical concerns, the setting had to be adapted and simplified for the main study. The pilot test and the methodological development will be reported in detail elsewhere.

#### Inclusion and exclusion criteria

All participants were being treated in the palliative care ward of the University Hospital Bonn in Germany. Patients had to be conscious and alert and understand the German language well enough to complete the questionnaires. They had to provide informed consent to participate in the study.

Patients were excluded if they were unconscious or severely fatigued. Potential test persons with multi-resistant infections could not provide saliva samples due to laboratory restrictions.

All included patients were randomized to intervention or control group using a simple randomization list (using the random number generation function in Excel). The study was not blinded nor allocation concealed as the ethics committee had requested to include information on the specific burden related to participation in the study for each group. One of the authors (LLD), who is a researcher in the Department of Palliative Medicine, but not involved in clinical care, enrolled and assigned the patients to one of the groups. The power calculation resulted in overall 240 patients to achieve a medium effect of *d* = 0.50 in the State-Trait-Cheerfulness Questionnaire (power = 0.70, Cohen’s *d*), with 120 in the intervention and 120 in the control group. Patients in the control and intervention group were tested on different days to avoid any inferences between the groups. The primary outcome was the mood of patients (State-Trait-Cheerfulness-Inventory). Secondary outcomes were burden of symptoms, distress, life satisfaction, and oxytocin level in saliva.

### Instruments

The set of questionnaires included the State-Trait-Cheerfulness-Inventory (STCI-T and -S) [10, 14], the Satisfaction with Life Scale (SWLS) [15], the stress thermometer [16], the Minimal Documentation System for patients in palliative care (MIDOS) [17, 18], and a few psychometric variables (age, gender, illness). The ECOG performance status was derived from the patient files [ECOG, 19]. The German self-rating version of all questionnaires was used.

Cheerfulness was measured using the STCI-T and -S [10, 14, 20, 21]. The STCI-T and -S, which are rated on 4-point Likert scale (from “strongly disagree” to “strongly agree”), consist of the three scales cheerfulness, seriousness, and bad mood, which are built from sum scores of 10 (STCI-T) and 6 (STCI-S) items, respectively. Mean values of 25.75 (*SD* 6.87) for cheerfulness, 24.28 (*SD* 6.03) for seriousness, and 15.20 (*SD* 6.31) for bad mood have been described in healthy subjects for the STCI-T [20]. The STCI-S evaluates the mood in the current situation, while the STCI-T investigates enduring personality traits [10, 14, 20, 21].

Symptom burden and well-being were assessed with the MIDOS [17, 18]. MIDOS is a short instrument with 8 items on physical and psychological symptoms and one item on general well-being, using categorical scales.

Life satisfaction was measured with the SWLS [15] with the sum score of 5 items, each rated on a 7-point Likert scale.

The distress thermometer consists of a scale from 0 to 10 where participants can mark their level of stress in the current situation [16].

Saliva samples were collected by a study nurse by having the patient chew on a cotton wool roll (Salivette® Sarstedt) for at least 60 s. Then the concentration of oxytocin in saliva [11] was analysed.

The researcher assisted the patients in completing the questionnaires. Depending on their performance level, she read the questions aloud or supervised independent completion.

### Intervention

The “humorous visit” was offered to the patients of the intervention group. Two professional hospital clowns who were dressed in the bright style of Mr. Bean (as clown outfits and the red nose seemed unsuitable for the setting) visited the patients one or two times. The training was performed by Laura Fernandez. The primary goal was to find the connection between the clown characters “Robert “ and”Lilly”, their joy, their humour, their differences, and their abilities. This was followed by exercises to “be in the moment”, “to get in touch”, and to find a playful or calm way from there on. Improvisation was one tool for training and to establish a trustful understanding between the clown actors. As both clown actors play instruments, making music together became not only an important part for the interventions, but also a nice warm-up for the clown actors to re-focus on their goals every week before the intervention. The intervention was based on McGhee’s humour habits programme, which was adapted by Falkenberg et al. [9] to a five-session training—memory of a funny episode during childhood (finding one’s preferred humour style), providing humour according to that style to the participant, finding humorous aspects in the current situation, producing humour, and applying humour in everyday life. The content of these 5 group sessions was condensed to two tailored humorous visits per patient. The coaches used various props (colourful cloths, a hand puppet, heating jacket tubes, musical instruments), but mostly they communicated and used their own and the patient’s imagination to build humorous interactions with the materials in the room (cushions, curtains, whiteboard, a wheelchair, etc.). Both coaches were educated as hospital clowns and play at least one instrument; one studied at a circus school and is a trained actress; the other studied at a clown school and is a certified social worker.

Entering the patient’s room, the humour coaches explored the mood of the patient and tried to find an adequate vibe to communicate. They asked a couple of questions concerning the biography and important life events to find out the patient’s preferred humour style. They then tried to find humorous aspects of the current situation using equipment in the room or making up a funny song about something the patient had mentioned. If the patients were still at the palliative care ward in the following week, they prepared a second visit focusing on aspects that were dear to the patient. As planning into the future is limited for patients in palliative care, they sometimes acted out unfulfilled wishes (such as a concert with songs of a specific singer or a cruise) in a caring and humorous manner.

The control group filled out the questionnaires twice and then provided saliva samples as well 1 day before the intervention group.

### Procedure

Data collection was performed according to the scheme displayed in Table 1. The control group was evaluated with the same routine except the interventions. The researcher documented field notes during the interventions, which were supplemented by questionnaires that the humour coaches completed after the humorous visits. The field note logs included start and end times and time stamps.

**Table 1:** Procedure of data collection intervention group

	Procedure (time)	Assessment instruments
Day 0	Briefing (10)	Informed written consent
Day 1	Assessment of psychological parameters (questionnaires) (15–25)	State-Trait-Cheerfulness (STCI-S and T), life satisfaction (SWLS), stress
	Assessment of medical parameters (5–10)	Burden of symptoms (MIDOS), oxytocin level, ECOG
	1st humour intervention (20–30)	Standardized non-participant observation (notes, “questionnaire” humour coaches, start and ending time)
	Assessment of the impact of the intervention (5–10)	STCI-S − oxytocin level
Day 3	Assessment of psychological parameters (questionnaires) (15–25)	STCI-S and T, SWLS, stress − MIDOS, oxytocin level
	2nd humour intervention (20–30)	Same as 1st humour intervention
	Assessment of the impact of the intervention (5–10)	STCI-S, SWLS, stress + MIDOS, oxytocin level

### Analyses

We implemented SPSS statistics for quantitative and MAXQDA for qualitative analyses. For pre- and post-workshop comparisons, *t*-tests were performed on mean values of the grouped data of all participants of the intervention and control group that completed the questionnaires. We included patients who had not more than two missing values in the main outcome variables STCI-S and SWLS in the evaluation. An ANOVA was used to compare pre-post values for both groups. We also compared means of the STCI-T, ECOG, and SWLS sum scores as well the MIDOS results of both groups to analyse potential differences in personality to agree to take part in a humour intervention.

An inductive-deductive approach was used to analyse the qualitative data. The inductively defined codes were condensed with additional codes until saturation was reached. The details of these analyses will be reported elsewhere.

## Results

### Sample

**Fig. 1:**
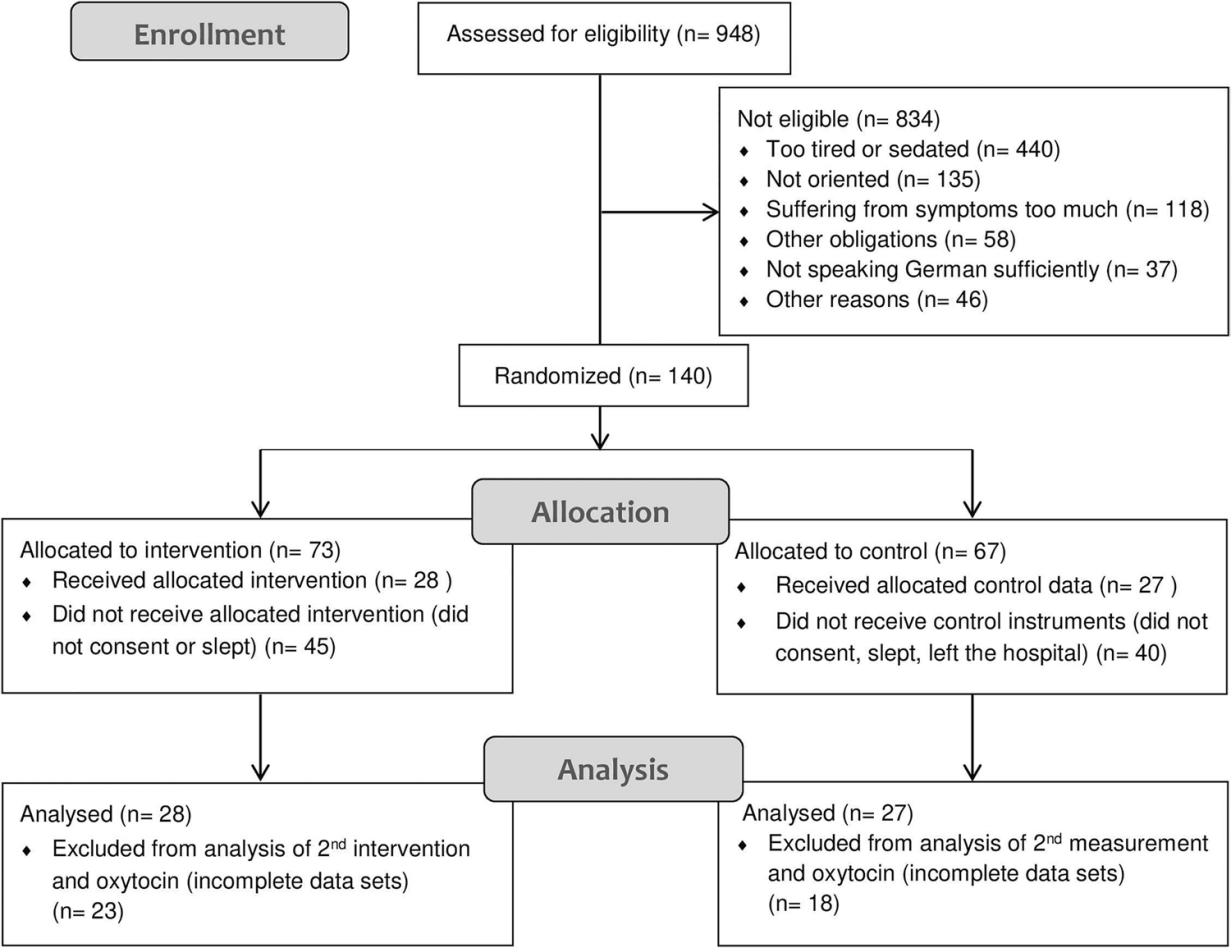
Flowchart patient recruitment

Overall 984 patients were scanned for eligibility from October 2017 to April 2019, and 140 patients were recruited for the study. However, only 55 patients completed the questionnaires and were included in the evaluation (27 were in the control group and 28 in the intervention group; see Fig. 1). Gender was well distributed with 27 women and 28 men (intervention group 16 ♀/12 ♂, control group 11 ♀/16 ♂). Age ranged from 29 to 99 years with a median of 64.48 (*SD* 14.09). All but 7 patients had an oncological diagnosis. ECOG performance status at admission was 2.91 (*SD* 0.95; min 0–max 4). In addition to the 55 patients included in the evaluation, another 68 patients received the humour intervention even though they were not able to complete the questionnaires. No patient reported adverse events or additional emotional burden from the humour intervention or data collection.

### Missing values

Only five of the 28 patients who received a second intervention were able to fill out the complete questionnaires again before and after the intervention to make an evaluation of quantitative data possible. Oxytocin in saliva could only be derived from 9 patients of the intervention and 9 of the control group, and thus, oxytocin data were not included in the analysis.

### Group comparisons

There were no significant differences in the pre-test scores for life satisfaction (*t* (48) = 0.70, *p* > 0.001) between intervention (*M* = 20.24, *SD* = 7.94) and control group (*M* = 18.72, *SD* = 7.24). Bad mood was slightly but not significantly higher in the control group (*t* (46) =  − 0.57) with mean values *M* = 22.50 (*SD* = 9.39) in the control and *M* = 21.13 (*SD* = 7.01) in the intervention group. The magnitude of the differences in the means (mean difference = 0.63, 95% *CI*: − 3.51 to 4.75) was very small for life satisfaction and also small for the nine other parameters that were investigated (see Table 2). The statistical parameters show that the intervention and control group were highly similar before the intervention in all investigated features. ANOVA analysis showed no significant differences in between groups (see Table 3).

**Table 2:** Pre-test group differences

				Test for equality of means		
		*M*	*SD*	*T*	*df*	*p*
Life satisfaction	Intervention	20.24	7.94	0.70	48	0.483
	Control	18.72	7.24			
Stress	Intervention	5.11	2.95	− 0.86	38	0.397
	Control	5.84	2.44			
Symptom burden	Intervention	19.41	6.28	− 0.32	30	0.751
	Control	20.07	5.12			
ECOG	Intervention	2.95	1.12	0.42	52	0.674
	Control	2.84	0.77			
Cheerfulness trait	Intervention	32.22	6.77	0.30	43	0.761
	Control	31.59	6.96			
Seriousness trait	Intervention	30.38	6.08	− 0.29	45	0.769
	Control	30.91	6.39			
Bad mood trait	Intervention	21.13	7.01	− 0.57	46	0.568
	Control	22.50	9.39			
Cheerfulness state	Intervention	11.53	4.81	0.04	53	0.969
	Control	11.48	4.62			
Seriousness state	Intervention	16.96	4.11	0.13	53	0.895
	Control	16.81	4.23			
Bad mood state	Intervention	13.24	5.56	1.45	53	0.152
	Control	11.22	4.67

**Table 3:** Univariate analysis for variances between intervention and control

		Squares	df	Mean Squares	*F*	*p*
Life satisfaction	Between groups	28.87	1	28.87	0.50	0.483
	In groups	2769.60	48	57.70		
Stress	Between groups	5.27	1	5.26	0.74	0.397
	In groups	272.46	38	7.16		
Symptom burden	Between groups	3.42	1	3.47	0.10	0.751
	In groups	1001.04	30	33.37		
ECOG	Between groups	0.16	1	0.16	0.18	0.674
	In groups	48.36	52	0.92		
Cheerfulness state	Between groups	0.03	1	0.03	0.00	0.969
	In groups	1180.55	53	22.27		
Seriousness state	Between groups	0.30	1	0.30	0.02	0.895
	In groups	927.04	53	17.48		
Bad mood state	Between groups	55.90	1	55.90	2.12	0.152
	In groups	1400.58	53	26.42		

In the control group, none of the investigated parameters changed significantly between pre- and post-measurement (see Table 4). For example, the score of state seriousness showed no significant change in between the pre- (*M* = 16.90, *SD* = 4.48) and post-measurements (*M* = 16.76, *SD* = 4.54), *t*(20) = 0.37. The mean change in the test scores was 0.13 with a 95% confidence interval ranging from − 0.63 to 0.91.

**Table 4:** Mean values pre- and post-measures in the control group

		*M*	*SD*	*t*	*df*	*p*
Life satisfaction	Before	19.78	6.92	0.00	17	1.000
	After	19.78	6.87			
Stress	Before	5.56	2.55	− 0.20	16	0.835
	After	5.59	2.51			
Symptom burden	Before	20.67	5.98	0.96	8	0.366
	After	20.11	6.40			
Cheerfulness	Before	11.14	4.33	0.76	20	0.452
	After	11.00	4.30			
Seriousness	Before	16.90	4.48	0.37	20	0.706
	After	16.76	4.54			
Bad mood	Before	11.00	4.62	− 1.27	20	0.219
	After	11.57	5.06			

As expected, the *t*-test for paired samples for the pre- and post-measures in the intervention group found four significant effects (see Table 5). The scores of distress, cheerfulness, seriousness, and bad mood changed significantly between pre- and post-measurements (Table 5).

**Table 5:** Mean values pre- and post-measures in the intervention group

		*M*	*SD*	*t*	*df*	*p*
Life satisfaction	Before	19.94	7.35	1.63	15	0.123
	After	18.56	7.09			
Stress	Before	5.55	2.90	2.40	10	0.037*
	After	3.41	2.63			
Symptom burden	Before	19.80	7.35	1.72	9	0.120
	After	18.30	5.89			
Cheerfulness	Before	11.49	4.23	− 4.06	19	0.001**
	After	15.80	5.40			
Seriousness	Before	16.45	4.60	2.90	19	0.009*
	After	13.10	4.02			
Bad mood	Before	13.35	5.51	3.11	19	0.006*
	After	9.95	4.43			

**p ≤ 0.001; *p ≤ 0.05.

### Qualitative data

Field notes were documented for all patients in the intervention group by the researcher. The field notes were coded and afterwards categorized into condition, contact, situation and life, expression of emotion, positive aspects, and symptoms. In the category condition, the code “deep breath” was coded most frequently. Frequent topics for contact were “participation”, “reception”, and “thank you/expression of gratitude”. “Reported memories” were predominant in situation and life. Expressions of emotion were very versatile, but signs of emotion were frequently coded. The category positive aspects included the highest number of codes including “smile”, “laugh”, “I like/that was great”, “joke”, and “applause”. Symptom codes were related to fatigue and exhaustion. During the coding of the data, new codes were added during the first half of the protocols, after which saturation occurred and the existing codes were sufficient for the analysis of the protocols. Exemplary quotes can be accessed in a table in the supplementary files.

## Discussions and conclusions

### Limitations

There was major attrition in this study, leading to many incomplete datasets and only very few patients that were treated according to protocol, even though we had shortened and simplified assessment instruments and intervention following a pilot testing. Most randomized patients did not consent to participate in the study due to feeling fatigued or being sedated, and most of those who participated were not available for a second humour intervention as they had been discharged or transferred to another place of care. Therefore an intention-to-treat analysis was not feasible. With almost zero questionnaire data available from these patients, imputation was not possible, and an elevation of *N* in the existing analyses would have distorted the standard error and painted an overly positive picture of the effect. We were not able to meet the power analyses calculated for the study plan with this inadequate sample size. Considering the high degree of attrition, we decided to stop the study after 18 months of recruitment. In consequence, we could only evaluate the results of the first humour intervention in this article in a smaller as planned sample. Ultimately, only 14% of the patients treated in the palliative care unit in the 18-month recruitment period were found to be eligible for the study, and only 39% of those patients (6% of the total patient number) participated and were included in the analysis. Oxytocin in saliva could only be sampled from 9 patients of each group and thus was not included in the analysis.

The control group did not receive an alternative intervention, due to feasibility reasons. This inactive control group setting creates the risk of performance bias. Lack of an active control also prevented adequate blinding and allocation concealment. These limitations may limit the transferability of the results. However, inclusion rates did not differ significantly between the intervention and control group, indicating a low risk of allocation bias. Methodologically, it would have been useful to set a cut-off value for cheerfulness to rule out bias by higher levels of cheerfulness in the persons who agreed to participate in the study. However, since levels of state cheerfulness did not show significant differences between intervention and control group, it can at least be assured that there was no bias due to allocation. The effect of cheerfulness on the willingness to participate might not be specific to humour interventions though, as a higher level of depression has been shown to limit the willingness to participate in any kind of study [22]. Thus, this limitation would not produce a specific bias of this study but rather seems to be a general phenomenon due to the elaborated precaution of persons with symptoms of depression.

The comparison of control and intervention group at the start of data collection showed that there was no significant difference between groups. Even though allocation was not concealed, these data seem to ensure comparability of both groups.

Ethical aspects of collecting complex data with severely ill patients at the end of their lives need to be discussed [23]. The SWLS questions for example were found to be distressing by more than half of the patients surveyed. Asking them how happy they are with their lives at the moment after receiving a palliative diagnosis seemed inappropriate.

In the original study plan, we had included a semi-structured interview for day 5 of the data collection. This was only possible in very few cases due to patient discharge, fatigue, and exhaustion of patients after the data collection and interventions.

### Discussion

Although we had already simplified the intervention after the pilot test and reduced the number of intervention appointments, still only a very small proportion of palliative care patients were eligible, and even fewer were able to provide sufficient data from the first intervention. This phenomenon of high attrition rates has been reported in palliative care previously [21, 24]. Attrition has even been identified as a major problem in palliative care research [24, 25]. We aimed to reduce attrition with a combination of patient-reported outcome questionnaires with physiological parameters [11, 26]. Using the level of oxytocin in saliva as biomarker has been evaluated critically in the past [27] because of its strong concentration fluctuations and potentially not measurable amount in saliva. The radioimmunoassay (RIA) method [28], which has been used in this study, can measure even very small amounts of oxytocin. However, even though saliva samples do not place a huge burden on patients, these assessments were possible only in a small minority of the study patients. Many patients suffered from xerostomia or were not able to chew on the swab for 60 s due to nausea. Half of the samples did not contain enough liquid for analysis.

Even though the analysis of variance did not show a significant effect of the intervention in the pre- and post-comparison, the comparison of pre- and post-data between the intervention and control group presented some promising results despite the small sample size. Whereas there were no significant changes in the control group, the perceived level of stress, seriousness, and bad mood were reduced in the intervention group, and cheerfulness increased. The positive effects of the humour intervention were supported in the qualitative analysis of the field notes. However, Bland and Altman [29] warned to use baseline as parameter for comparison. Recruitment of a bigger sample would be desirable in order to evaluate analysis of variance in the pre- and post-data between groups.

In the intervention group, life satisfaction was slightly lower after the intervention, in contrast to the positive findings for level of stress, seriousness, bad mood, and cheerfulness. A possible explanation could be that completing the questionnaires might have had a negative effect on patients’ life satisfaction. This effect has been reported in literature in the past [30], and there has been even a questionnaire created to measure negative effects [31]. However, neither the intervention group nor the control group showed this effect. More research is needed to evaluate this discrepancy.

Our study confirmed that short and simple assessment instruments are a mandatory precondition for palliative care research. However, evaluation of the effectiveness of humour interventions in patients with far-advanced disease might require proxy-reported instead of patient-reported outcome measures and observational assessment instruments as well as suitable biomarkers. Again, problems with sampling have to be considered, such as the high frequency of dry mouth or swallowing problems which can interfere with saliva sampling.

### Conclusion

Major problems with attrition led to a smaller as planned sample size in our intervention study. However, we found some promising results for a positive effect of the humour interventions for patients in palliative care. Further research could be planned for the outpatient and home care setting, recruiting patients less advanced in the disease trajectory and thus with less physical or cognitive impairment compared to those on a palliative care unit. However, standardized training of clowns for this kind of humour intervention would be a necessary prerequisite for such a roll-out.

## Supplementary Information

Below is the link to the electronic supplementary material.

Supplementary file1 (DOCX 106 KB)

Supplementary file2 (ODT 7 KB)

## Data Availability

Due to privacy regulations considering data from patients being treated in a German university hospital, we cannot provide the data to others. The ethics committee also demanded a paragraph stating that the data will only be used by us in pseudonymized form for the publication of the results of our study.
