# Supplementary material for: Humour interventions for patients in palliative care—a randomized controlled trial
Source: Support Care Cancer. 2023 Feb 13;31(3):160. doi: 10.1007/s00520-023-07606-9 (PMC9925513; doi:10.1007/s00520-023-07606-9)
Supplement: Supplementary file 1 — Supplementary file1 (DOCX 106 KB) [file 520_2023_7606_MOESM1_ESM.docx]

**Prob-Nr. Datum:**

**Fragebögen**

Die folgenden Aussagen beziehen sich auf Ihre **augenblickliche** Stimmung. Versuchen Sie bitte, Ihre momentane, gefühlsmäßige Stimmung zu beschreiben, indem Sie eine von vier Antwortalternativen ankreuzen. Die vier Alternativen lauten:

[1] ..... trifft gar nicht zu

[2] ..... trifft eher nicht zu

[3] ..... trifft etwas zu

[4] ..... trifft sehr zu

Bitte bearbeiten Sie alle Aussagen!

|  | trifft gar nicht zu | trifft eher nicht zu | trifft etwas zu | trifft sehr zu |
| --- | --- | --- | --- | --- |
| 1. Ich habe schlechte Laune. | ❑ | ❑ | ❑ | ❑ |
| 2. Ich bin ausgelassen. | ❑ | ❑ | ❑ | ❑ |
| 3. Ich bin eher auf Ernstes eingestellt. | ❑ | ❑ | ❑ | ❑ |
| 4. Ich bin betrübt. | ❑ | ❑ | ❑ | ❑ |
| 5. Mir gehen jetzt wichtige Dinge durch den Kopf. | ❑ | ❑ | ❑ | ❑ |
| 6. Ich bin fröhlich. | ❑ | ❑ | ❑ | ❑ |
| 7. Ich bin nachdenklich. | ❑ | ❑ | ❑ | ❑ |
| 8. Ich bin in gehobener Stimmung. | ❑ | ❑ | ❑ | ❑ |
| 9. Ich bin deprimiert. | ❑ | ❑ | ❑ | ❑ |
| 10. Meine Geisteslage ist durch Ernsthaftigkeit geprägt. | ❑ | ❑ | ❑ | ❑ |
| 11. Ich bin genervt. | ❑ | ❑ | ❑ | ❑ |
| 12. Ich bin heiter. | ❑ | ❑ | ❑ | ❑ |
| 13. Mir ist zum Lachen zumute. | ❑ | ❑ | ❑ | ❑ |
| 14. Ich bin traurig. | ❑ | ❑ | ❑ | ❑ |
| 15. Mir ist nicht nach Oberflächlichem zumute. | ❑ | ❑ | ❑ | ❑ |
|  | trifft gar nicht zu | trifft eher nicht zu | trifft etwas zu | trifft sehr zu |
| 16. Ich bin lustig. | ❑ | ❑ | ❑ | ❑ |
| 17. Ich bin ernst. | ❑ | ❑ | ❑ | ❑ |
| 18. Ich fühle mich unwohl. | ❑ | ❑ | ❑ | ❑ |

STCI_518

Bitte überprüfen Sie abschließend noch einmal, ob Sie **alle** Aussagen bearbeitet haben.

**Bitte kreuzen Sie bei den folgenden 5 Aussagen an, inwieweit Sie diesen zustimmen.**

|  | stimme  völlig zu | stimme  zu | stimme  eher zu | weder/  noch | stimme  eher  nicht zu | stimme  nicht zu | stimme  überhaupt  nicht zu |
| --- | --- | --- | --- | --- | --- | --- | --- |
| In den meisten Bereichen entspricht mein Leben meinen Idealvorstellungen. | ❑ | ❑ | ❑ | ❑ | ❑ | ❑ | ❑ |
| Meine Lebensbedingungen sind ausgezeichnet. | ❑ | ❑ | ❑ | ❑ | ❑ | ❑ | ❑ |
| Ich bin mit meinem Leben  zufrieden. | ❑ | ❑ | ❑ | ❑ | ❑ | ❑ | ❑ |
| Bisher habe ich die wesentlichen Dinge erreicht, die ich mir für mein Leben wünsche. | ❑ | ❑ | ❑ | ❑ | ❑ | ❑ | ❑ |
| Wenn ich mein Leben noch  einmal leben könnte, würde ich kaum etwas ändern. | ❑ | ❑ | ❑ | ❑ | ❑ | ❑ | ❑ |

SWLS

Die folgenden Aussagen beziehen sich auf Ihre Stimmungen und Ansichten **im Allgemeinen**. Versuchen Sie bitte anhand der folgenden Aussagen Ihre **üblichen** Verhaltensweisen und Einstellungen so gut wie möglich zu beschreiben, indem Sie eine von vier Antwortmöglichkeiten ankreuzen. Die vier Alternativen lauten:

(1) … trifft gar nicht zu

(2) … trifft eher nicht zu

(3) … trifft etwas zu

(4) … trifft sehr zu

Beispiel: Ich bin ein aktiver Mensch. (1) (2) (3) (4)

Wenn diese Aussage Sie sehr treffend beschreibt – d.h. wenn Sie im Allgemeinen ein aktiver Mensch sind – kreuzen Sie bitte (4)

an. Trifft diese Aussage auf Sie dagegen überhaupt nicht zu, dann kreuzen Sie bitte (1) an.

Sollten Sie einmal Schwierigkeiten haben, zu einer Frage Ihre Antwort zu finden, kreuzen Sie bitte die am ehesten zutreffende an. Kreuzen Sie bitte zu jeder Aussage eine der vier Antwortmöglichkeiten an, und lassen Sie keine Frage aus.

|  | Trifft gar nicht zu | trifft eher nicht zu | trifft etwas zu | trifft sehr zu |
| --- | --- | --- | --- | --- |
| 1 Im Gespräch vermeide ich bewusst Übertreibungen, Ausschmückungen oder Doppeldeutigkeiten, da sie nichts zur Aussage beitragen. | ❑ | ❑ | ❑ | ❑ |
| 2 Mein Alltag bietet mir oft Anlass zum Lachen. | ❑ | ❑ | ❑ | ❑ |
| 3 Meine Mitmenschen haben häufig einen Grund, mich zu fragen, ob mir „eine Laus über die Leber“ gelaufen sei. | ❑ | ❑ | ❑ | ❑ |
| 4 Es kann vorkommen, dass ich für längere Zeit in einer betrübten Stimmung bin. | ❑ | ❑ | ❑ | ❑ |
| 5 Ich bin auf größtmögliche Korrektheit bedacht. | ❑ | ❑ | ❑ | ❑ |
| 6 Es gibt Tage, an denen ich mich innerlich leer fühle. | ❑ | ❑ | ❑ | ❑ |
| 7 Ich neige dazu, weit im Voraus zu planen und mir langfristige Ziele zu stecken. | ❑ | ❑ | ❑ | ❑ |
| 8 Ich bin ein fröhlicher Typ. | ❑ | ❑ | ❑ | ❑ |
| 9 Bei allem, was ich tue, bedenke ich stets die möglichen Folgen und vergleiche alle möglichen Vor- und Nachteile sorgfältig. | ❑ | ❑ | ❑ | ❑ |
| 10 Auch ohne besonderen Anlass bin ich häufig verstimmt. | ❑ | ❑ | ❑ | ❑ |

|  | trifft gar nicht zu | trifft eher nicht zu | trifft etwas zu | trifft sehr zu |
| --- | --- | --- | --- | --- |
| 11 Es fällt mir leicht, gute Laune zu verbreiten. | ❑ | ❑ | ❑ | ❑ |
| 12 Ich lache gerne und viel. | ❑ | ❑ | ❑ | ❑ |
| 13 Einer meiner Grundsätze lautet: „Erst die Arbeit, dann das Vergnügen!“. | ❑ | ❑ | ❑ | ❑ |
| 14 Es gibt häufig Tage, an denen der Spruch „ich bin mit dem falschen Fuss aufgestanden“ gut meine Gemütsverfassung beschreibt. | ❑ | ❑ | ❑ | ❑ |
| 15 Lachen wirkt auf mich sehr ansteckend. | ❑ | ❑ | ❑ | ❑ |
| 16 Im Kontakt mit anderen fällt mir immer wieder auf, dass ich viel gründlicher über verschiedene Sachen nachgedacht habe. | ❑ | ❑ | ❑ | ❑ |
| 17 Ich bin ein lustiger Mensch. | ❑ | ❑ | ❑ | ❑ |
| 18 Da Entscheidungen Konsequenzen nach sich ziehen, vermeide ich es, Angelegenheiten oberflächlich zu behandeln bzw. leichtfertige Entschlüsse zu fassen. | ❑ | ❑ | ❑ | ❑ |
| 19 Ich habe die Erfahrung gemacht, dass an dem Sprichwort „Lachen ist die beste Medizin“ wirklich etwas dran ist. | ❑ | ❑ | ❑ | ❑ |
| 20 Ich denke oft: „Mensch, lasst mich heute bloß in Ruhe!“ | ❑ | ❑ | ❑ | ❑ |
| 21 Auch scheinbare Kleinigkeiten bedürfen einer ernsthaften und verantwortungsvollen Behandlung. | ❑ | ❑ | ❑ | ❑ |
| 22 Ich bin häufig in missmutiger Stimmung. | ❑ | ❑ | ❑ | ❑ |
| 23 Ich bin leicht zum Lachen zu bringen. | ❑ | ❑ | ❑ | ❑ |
| 24 Verglichen mit anderen kann ich ganz schön mürrisch und griesgrämig werden. | ❑ | ❑ | ❑ | ❑ |
| 25 Ich tue nur Dinge, die einen gewissen Sinn ergeben; alles andere ist unnütze Zeitverschwendung und zwecklos. | ❑ | ❑ | ❑ | ❑ |
|  | trifft gar nicht zu | trifft eher nicht zu | trifft etwas zu | trifft sehr zu |
| 26 Ich bin manchmal auch ohne Grund ganz traurig. | ❑ | ❑ | ❑ | ❑ |
| 27 Ich mag Menschen, die Überlegung und Sachlichkeit ausstrahlen. | ❑ | ❑ | ❑ | ❑ |
| 28 Ich habe ein sonniges Gemüt. | ❑ | ❑ | ❑ | ❑ |
| 29 Ich bin häufig niedergeschlagen. | ❑ | ❑ | ❑ | ❑ |
| 30 Die kleinen Missgeschicke des Alltags finde ich oft amüsant, selbst wenn sie mich betreffen. | ❑ | ❑ | ❑ | ❑ |

STHI_T30

Alle Aussagen bearbeitet?


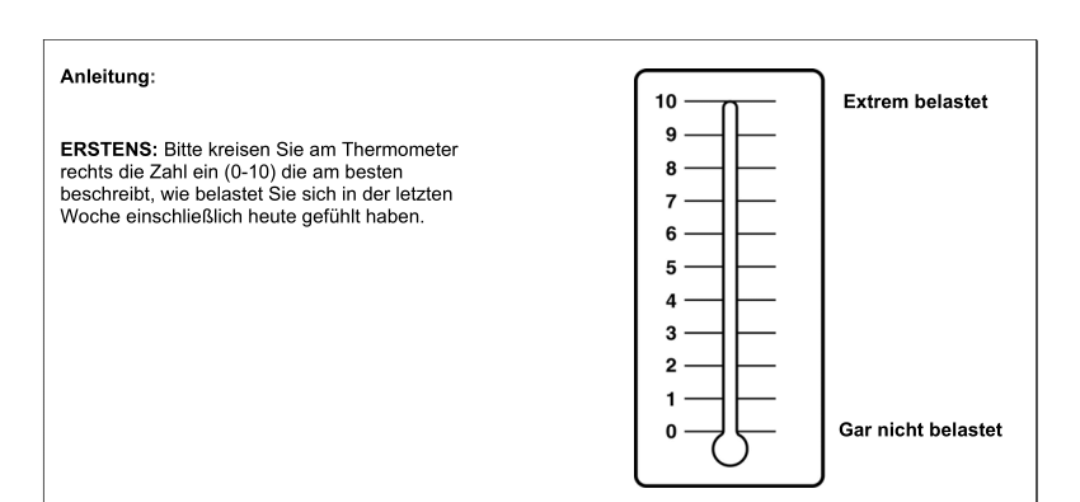


Sehr geehrte Patientin, sehr geehrter Patient,

Sie kennen Ihre Situation selber am Besten. Darum bitten wir Sie, diesen Bogen sorgfältig auszufüllen und die Aussagen so anzukreuzen, wie Sie sie im Augenblick bei sich selber wahrnehmen.

| **1.** Bitte kreuzen Sie an, wie **stark** heute **Ihre Beschwerden** sind. | | | | | | | | | | | | |
| --- | --- | --- | --- | --- | --- | --- | --- | --- | --- | --- | --- | --- |
| **Schmerz** | | | ❑ keine | | | ❑ leichte | | ❑ mittlere | | | ❑ starke Schmerzen | |
| Übelkeit | | | ❑ keine | | | ❑ leichte | | ❑ mittlere | | | ❑ starke Übelkeit | |
| Erbrechen | | | ❑ kein | | | ❑ leichtes | | ❑ mittleres | | | ❑ starkes Erbrechen | |
| Luftnot | | | ❑ keine | | | ❑ leichte | | ❑ mittlere | | | ❑ starke Luftnot | |
| Verstopfung | | | ❑ keine | | | ❑ leichte | | ❑ mittlere | | | ❑ starke Verstopfung | |
| Schwäche | | | ❑ keine | | | ❑ leichte | | ❑ mittlere | | | ❑ starke Schwäche | |
| **Appetitmangel** | | | ❑ kein | | | ❑ leichter | | ❑ mittlerer | | | ❑ starker Appetitmangel | |
| **Müdigkeit** | | | ❑ keine | | | ❑ leichte | | ❑ mittlere | | | ❑ starke Müdigkeit | |
| Depressivität | | | ❑ keine | | | ❑ leichte | | ❑ mittlere | | | ❑ starke Depressivität | |
| Angst | | | ❑ keine | | | ❑ leichte | | ❑ mittlere | | | ❑ starke Angst | |
| Andere: | | | ❑ keine | | | ❑ leichte | | ❑ mittlere | | | ❑ starke | |
| Andere: | | | ❑ keine | | | ❑ leichte | | ❑ mittlere | | | ❑ starke | |
| **2.** Bitte kreuzen Sie an, wie Sie sich heute **fühlen**: | | | | | | | | | | | | |
| **Befinden** | ❑ sehr schlecht | | | ❑ schlecht | | | ❑ mittel | | | ❑ gut | | ❑ sehr gut |
| **3.** Bemerkungen: | | | | | | | | | | | | |
|  |  | | |  | | |  | | |  | |  |
| **4.** Selbsterfassung **nicht möglich** wegen: | | | | | | | | | | | | |
| ❑ Sprachproblemen | | ❑ Schwäche | | | ❑ Kognitiven Störungen | | | | ❑ Patient lehnt ab | | | ❑ keine Zeit |

MIDOS
