# Supplementary material for: Humour interventions for patients in palliative care—a randomized controlled trial
Source: Support Care Cancer. 2023 Feb 13;31(3):160. doi: 10.1007/s00520-023-07606-9 (PMC9925513; doi:10.1007/s00520-023-07606-9)
Supplement: Supplementary file 2 [file 520_2023_7606_MOESM2_ESM.odt]

Table: Examples from most frequent codes in qualitative data

| Code | Exemplary quotes |
| --- | --- |
| expression of appeal | “Really very beautiful.”; “You are in ‘Recall’.”; “Yes! That was amazing.”; “It was great.”; |
| applause | clapping or knocking on the little table |
| smile | variations and broadness of smiles |
| laughter | variations and length of laughter |
| good memories | “We watched the semi-finals of the handball World Championships.”; “I’ve always sung.”; “I was a beauty.”; “I celebrated my silver wedding.”; “I had great friends. I am so thankful they took such a liking in me.” |
| production of humour | “Here you go, he´s on a leash.” (after a humourist tied a walker onto a rope); “Marx is dead, Mao is dead and I feel quite sick, too.”; “This is a similarly funny book.”; “You rehearsed a little, at least.”; “The voice recorder touched her forehead.” |
| gratitude | very similar expressions of gratitude – “Thank you.”; “Thank you very much.” |
| approval | different expressions of approval: “Yes.”; nodding; “Yes, that’s right.”; “I think so, too.”; “Good.” |
| fatigue | signs of fatigue: closing his/her eyes; “I am getting tired.” |

*translated verbatim from German, sorted by frequency of occurrence
